# Efficacy of autologous bone marrow buffy coat grafting combined with core decompression in patients with avascular necrosis of femoral head: a prospective, double-blinded, randomized, controlled study

**DOI:** 10.1186/scrt505

**Published:** 2014-10-14

**Authors:** Yuanchen Ma, Tao Wang, Junxing Liao, Honglin Gu, Xinpeng Lin, Qing Jiang, Max K Bulsara, Minghao Zheng, Qiujian Zheng

**Affiliations:** Division of Orthopaedic Surgery, Department of Surgery, Guangdong General Hospital, Guangdong Academy of Medical Sciences, 106 Zhongshaner Rd, Guangzhou, Guangdong 510080 P.R. China; Centre for Orthopaedic Translational Research, School of Surgery, The University of Western Australia, M Block, QE2 Medical Centre, Monash Ave, Nedlands, WA 6009 Australia; The Centre for Diagnosis and Treatment for Joint Disease, Drum Tower Hospital, The Affiliated Hospital to Medical School of Nanjing University, 321 Zhongshan Rd, Nanjing, Jiangsu 210008 P.R. China; Institute for Health Research, University of Notre Dame, 19 Mouat St, Fremantle, WA 6959 Australia

## Abstract

**Introduction:**

Avascular necrosis of femoral head (ANFH) is a progressive disease that often leads to hip joint dysfunction and even disability in young patients. Although the standard treatment, which is core decompression, has the advantage of minimal invasion, the efficacy is variable. Recent studies have shown that implantation of bone marrow containing osteogenic precursors into necrotic lesion of ANFH may be promising for the treatment of ANFH.

**Methods:**

A prospective, double-blinded, randomized controlled trial was conducted to examine the effect of bone-marrow buffy coat (BBC) grafting combined with core decompression for the treatment of ANFH. Forty-five patients (53 hips) with Ficat stage I to III ANFH were recruited. The hips were allocated to the control group (core decompression + autologous bone graft) or treatment group (core decompression + autologous bone graft with BBC). Both patients and assessors were blinded to the treatment options. The clinical symptoms and disease progression were assessed as the primary and secondary outcomes.

**Results:**

At the final follow-up (24 months), there was a significant relief in pain (*P* <0.05) and clinical joint symptoms as measured by the Lequesne index (*P* <0.05) and Western Ontario and McMaster Universities Arthritis Index (*P* <0.05) in the treatment group. In addition, 33.3% of the hips in the control group have deteriorated to the next stage after 24 months post-procedure, whereas only 8% in the treatment group had further deterioration (*P* <0.05). More importantly, the non-progression rates for stage I/II hips were 100% in the treatment group and 66.7% in the control group.

**Conclusion:**

Implantation of the autologous BBC grafting combined with core decompression is effective to prevent further progression for the early stages of ANFH.

**Trial registration:**

ClinicalTrials.gov identifier NCT01613612. Registered 13 December 2011.

## Introduction

Avascular necrosis of femoral head (ANFH) is a progressive disease that often leads to hip joint dysfunction and thus disability in young patients. It is often initiated by insufficient blood supply to the femoral head, leading to increased intraosseous pressure and consequently causing subchondral plate collapse and osteoarthritis [1]. Total hip replacement is the common option for terminal ANFH; however, owing to poor prosthetic durability, it is not preferable for young patients [1]. Early intervention prior to subchondral bone collapse would thus be critical to preserve joint structure and function, which may postpone the need of total hip replacement in these patients. Conservative techniques, such as core decompression and avascular or vascularized bone grafting, have been used for the treatment of early-stage ANFH and shown good to moderate results [2–4]. Core decompression is the most common procedure as it is of minimal invasion. However, the efficacy of this technique has been variable. The clinical failure rate of this technique reached 30% in early-stage (Ficat stage I/II) patients [3, 5]. Vascularized or non-vascularized bone-grafting provides a better subchondral support and has been proven to be successful in reserving hip joint function and postponing the time for hip joint replacement [6]. However, the technique is more invasive and has complications such as donor site morbidity and nerve palsy. Thus, it is of importance to seek minimal invasive approaches with better clinical outcomes for the treatment of ANFH.

Accumulating evidence indicated that ANFH is associated with a decreased pool of osteoprogenitor cells in the bone marrow of femoral head [7–10]. Previous studies have shown that implantation of bone marrow containing osteogenic precursors into necrotic lesion of ANFH may be beneficial. Indeed, three out of four ANFH patients who received skeletal stem cell and milled-allograft bone therapy remained asymptomatic at 22 to 44 months’ follow-up [11], indicating that the efficacy of using skeletal bone marrow cells is promising for the treatment of ANFH. However, these studies are only at the proof-of-concept stage and require further clinical investigation [12, 13]. In addition, the use of allograft bone may have potential side effects such as virus infection for the treated patients.

Therefore, to minimize potential side effects as well as improve clinical outcomes, this study aims to combine minimal invasive core decompression technique, autologous bone graft from the affected femoral head, and bone-marrow buffy coat (BBC) implantation to investigate the clinical efficacy of this strategy on patients with Ficat stage I to III ANFH.

## Materials and methods

### Patients

Eligible patients were recruited from people administrated in the Department of Orthopedic Surgery (name of institution blinded) from June 2009 to Octorber 2010. All participants had plain radiograph of the hips at the anteroposterior and frog-leg lateral positions and magnetic resonance imaging [14]. Diagnosis of ANFH was made on the basis of clinical history and the presence of lesion in plain radiograph. Inclusion criteria were age between 18 to 55 years; notable pain in the hip; and normal, minor, or mixed osteopenia or the presence of crescent sign in plain radiograph. Patients with confirmed diagnosis of osteonecrosis in another joint and one or more associated risk factors were also recruited in the study. Exclusion criteria included the following: age of less than 18 or older than 55, end stage of ANFH with evidence of secondary degenerative changes, pregnancy, history of femoral head/neck fracture, tumor, or any other pathology; having previously received surgical/invasive intervention on hip, including core decompression, bone graft implantation, titanium implantation, and osteotomy; having received steroid treatment in the last 6 months; having systemic disorders such as diabetes, rheumatoid arthritis, and hepatitis; and incapability to understand or follow instructions. The study protocol was approved by the Guangdong General Hospital Human Ethical Committee, and informed consent was obtained from the participants. The trial protocol was submitted to ClinicalTrials.gov, and the trial registration number is NCT01613612.

### Study design

The study is a randomized, participant- and outcome assessor-blinded, controlled trial. After being determined eligible for the study, the participants were provided with informed consent, had a baseline assessment, and were subjected to randomization. A computer using block randomization of 10 hips was used to generate a randomization schedule. The hips were thus randomly allocated to receive core decompression + autologous bone graft or core decompression + autologous bone graft with BBC. All patients were blinded to treatment options. The surgeon performing the procedure was blinded to the patients’ clinical presentation but informed of the procedures of core decompression with or without BBC implantation. Patients with bilateral ANFH may receive different treatments on both hips at the same day. Outcome assessors were blinded to group assignment.

### Power calculation

The power of both groups was determined by using the results from previous publication [15]. At least 20 patients per group are necessary to measure the difference by using the Wilcoxon-Mann-Whitney test with a bilateral alpha of 0.017 and a power of 80%. To compensate for the patients lost to follow-up, we included a minimum of 26 patients in each group.

### Core decompression

**Figure 1:**
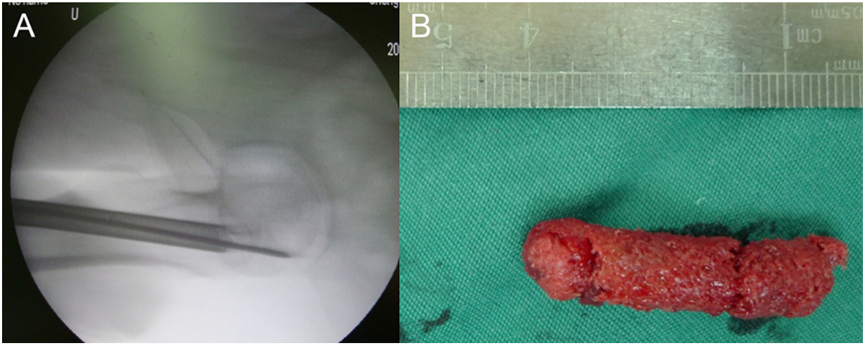
**Image of operation procedure. (A)** Radiography of the femoral head at the time of the operation. The Kirschner wire and 10-mm diameter trephine were driven into the mid-line of the trochanter by a cannulated drill under C-arm x-ray guidance toward the necrotic area. **(B)** A 3.5-cm long cylindrical bone was taken from the trochanter through the trephine and used for bone graft to enriched bone marrow incorporation.

The patients were positioned supine on the operation table and anesthetized by continuous epidural anesthesia. For core decompression of the femoral head, a 1.5-cm incision was made over the skin and the fascia on the lateral aspect the thigh at the level of the greater trochanter. A Kirschner wire (k-wire) was driven into the mid-line of the trochanter by a cannulated drill under C-arm x-ray guidance, pointing toward the necrotic area. The tip of the k-wire was placed at the subchondral bone region with a distance of 2 to 3 mm from the articular cartilage (Figure 1A). A 10-mm diameter trephine was placed through the k-wire and driven toward the necrotic site. A cylinder of bone from the femoral neck and head was obtained and used for bone marrow grafting (Figure 1B). The necrotic tissue in the femoral head was removed by using the bone curette.

### Bone marrow aspiration and bone-marrow buffy coat implantation

After the patient received continuous epidural anesthesia, the pelvic bone was punctured from the superior posterior iliac spine with the bone marrow aspiration needle (Biomid; Gallini Medical Devices, Mantova, Italy). The needle was rotated back and forth to achieve penetration through the cortical bone. A 50-mL heparinized syringe was connected to the bone marrow aspiration needle and used to harvest the bone marrow. The patients who received single core decompression underwent the same procedure except that no bone marrow was aspirated from the iliac crest. To obtain the BBC with enriched nucleated cells, bone marrow was centrifuged at 1500 revolutions per minute for 10 minutes in a bench-top centrifuge with a sterilized chamber. After centrifuge, the bone marrow was separated into three phases. The blood serum from the superficial layer was removed, and the BBC from the interface containing enriched bone marrow cells was collected by a sterilized transfer pippet and carefully loaded onto the porous cylindrical bone drop by drop. A total of 1 mL of BBC was isolated from the suspension, and 10 μL of BBC was kept for cell counting after the surgery. The average bone marrow cells loaded to the cylindrical bone were approximately 3 × 10^9^ nucleated cells. To allow the cells to anchor on the bone surface, the BBC was seeded on the cylindrical bone for 2 minutes before implantation. The bone graft was inserted into the necrotic region through the trephine. C-arm x-ray was used to ensure that the bone graft was placed into the necrotic site. For the patients who received single core decompression, the cylindrical bone was inserted into the necrotic region without the buffy coat. After surgery, the patients were instructed to remain non-weight-bearing for 4 weeks. Surgical complications, such as bone marrow harvest site infection, surgical site infection, and breeding, were monitored after operation.

### Outcome measurement

The blinded assessor evaluated all participants at baseline and at 3 and 24 months postoperatively. Baseline demographic information, including age, sex, etiological factors, presurgical Ficat stage of ANFH, and location of defect, was collected. Ficat stage of ANFH and the location of necrotic lesion in each hip were determined by plain x-ray radiograph and magnetic resonance imaging. The primary outcomes were the average pain on movement and function of hip. The pain was assessed by visual analogue scale (VAS). The function of hip was evaluated by using Lequesne algofunctional index and Western Ontario and McMaster Universities Arthritis Index (WOMAC) osteoarthritis scoring [15].

Secondary outcome was the improvement of disease progression determined by the stage of osteonecrosis and the status of subchondral bone collapse at the time of the final follow-up. The patients were followed for up to 24 months. Anteroposterior and frog-leg lateral radiographs were taken at the time of each clinical assessment. Magnetic resonance images of the affected hip were made at baseline and at the last follow-up. Radiographic progression of the ANFH was determined according to the Ficat and alert classification system. All radiographs were analyzed by the same blinded assessor who did not participate in the treatment.

### Statistical analysis

Variables are described as the mean and the standard deviation of the mean. The statistical significance of demographic structure, pre-operative evaluation, and clinical outcomes between two groups were determined by the Wilcoxon-Mann-Whitney test. The paired *t* test was used to compare the clinical assessment before and after operation. To account for multiple joints in the same patient, a linear mixed model was used to test for within-group differences. The significance for all tests was defined as a *P* value of less than 0.05.

## Results

### General information and patient flow chart

**Figure 2:**
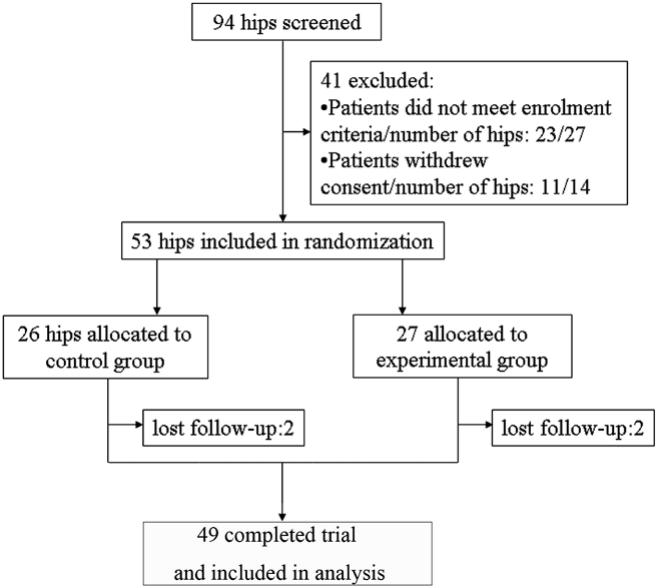
**Flow chart of patient enrollment and randomization.**

Ninety-four hips (77 patients) were screened for eligibility, and 41 hips (34 patients) were excluded. Finally, 45 patients with 53 hips were recruited and randomly assigned into two groups. Four patients with four hips were lost to follow-up at 3 months. Forty-nine hips (for 43 patients) have completed the clinical trial and were included in the final analysis (Figure 2). The demographic data and baseline characteristic of the hips are listed in Table 1. At the basal level, there was no significant difference of the stages of ANFH, etiological factors of the disease, the age of patients, and the clinical symptoms between two groups. Seven patients were bilaterally affected by ANFH. Eight hips had Ficat stage I osteonecrosis, 32 hips had Ficat stage II osteonecrosis, and 10 hips had stage III osteonecrosis. Twenty-six patients (29 hips) had a history of corticosteroid therapy, and seven patients (eight hips) had alcohol-induced ANFH. Twelve patients were idiopathic. The average age of the patients was 35 ± 9.8 years. There were 33 anterosuperior lesions at the weight-bearing sites and 16 lesions at the non-weight-bearing sites when the patients stood upright. All of the patients had reported notable pain in one or both hips. The hips in the control group were treated with single core decompression and implantation of autologous bone graft, and the treatment group was processed to core decompression and implantation of autologous bone grafted with enriched autologous bone-marrow nucleated cells. Twenty-two patients had bilateral ANFH. Among those, 10 patients (20 hips) received core decompression with/without BBC on both hips, and 12 patients received total hip replacement on one side of the hip for the treatment of end-stage osteonecrosis and core decompression with/without BBC on the other side for early osteonecrosis. Overall, 49 hips were available for final analysis. No complication was observed in the patients during or after the core decompression with/without BBC grafting.

**Table 1:** **Demographic data of the participants and baseline characteristics of the hips**

	Control	BBC implantation	***P*** value
**Number of patients**	18	21	
**Age, years (average ± SD)**	34.78 ± 11.48	35.60 ± 8.05*	0.99
<25	3	1	
25-34	7	9	
35-44	5	7	
45-54	2	4	
55+	1	0	0.61
**Sex**			
Male	13	15	
Female	5	6	0.96
**Etiogenic**			
Steroid	13	13	
Alcohol	3	4	
Idiopathic	6	6	
**Number of joints**	**24**	**25**	
Ficat I	4	3	
Ficat II	15	17	
Ficat III	5	5	
**Location of defect**			
Weight-bearing	6	9	
Non-weight-bearing	18	16	

*P =0.99. BBC, bone-marrow buffy coat.

### Primary outcomes of clinical symptom improvement

**Figure 3:**
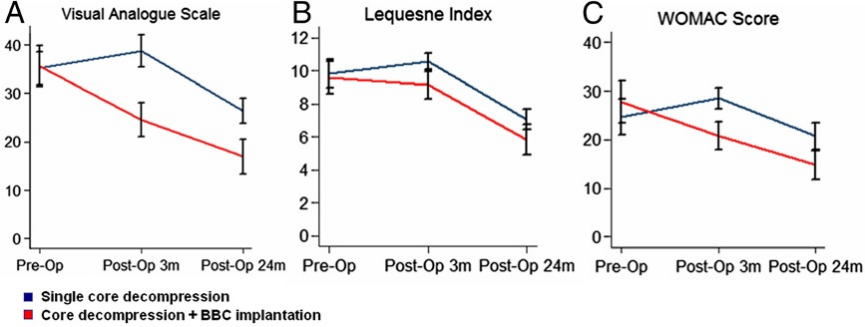
**A comparison between the control group and bone-marrow buffy coat (BBC) group of the visual analogue scale (A), the Lequesne index (B) and the Western Ontario and McMaster Universities Arthritis Index (WOMAC) score (C) at the pre-operation, 3 months post-operation, and 24 months post-operation.** The results were present as mean ± standard error of the mean. Asterisk (*) indicated a significant difference compared with the baseline; pound sign (#) indicated a significant difference compared with the control group.

In the control group with core decompression only, there is a slight increase of VAS from 35.21 ± 3.41 mm at the basal level to 38.75 ± 3.27 mm at 3 months after operation, but this decreased to 26.46 ± 2.60 mm at 24 months’ follow-up (*P* =0.007) (Figure 3A). In contrast, in the group of patients with core decompression combined with BBC, the level of pain measured by VAS was significantly decreased at 24 months after surgery. The mean value of VAS was significantly decreased from 35.58 ± 4.21 mm at the basal level to 24.62 ± 3.50 mm at 3 months (*P* <0.0.001) and 16.92 ± 3.66 mm (*P* <0.001) at the final follow-up (Figure 3A). Patients treated with core decompression combined with BBC also have a significant improvement in the joint symptoms, as evidenced by the Lequesne index and WOMAC score. The mean of Lequesne index decreased from 9.58 ± 0.99 at the basal level to 5.83 ± 0.93 (*P* <0.001) after 24 months in the BBC grafted group (Figure 3B). In addition, the average scores of WOMAC were 27.77 ± 4.23 at baseline and 14.81 ± 2.99 (*P* <0.001) at the last follow-up (Figure 3C).

### Secondary outcome of disease progression

**Figure 4:**
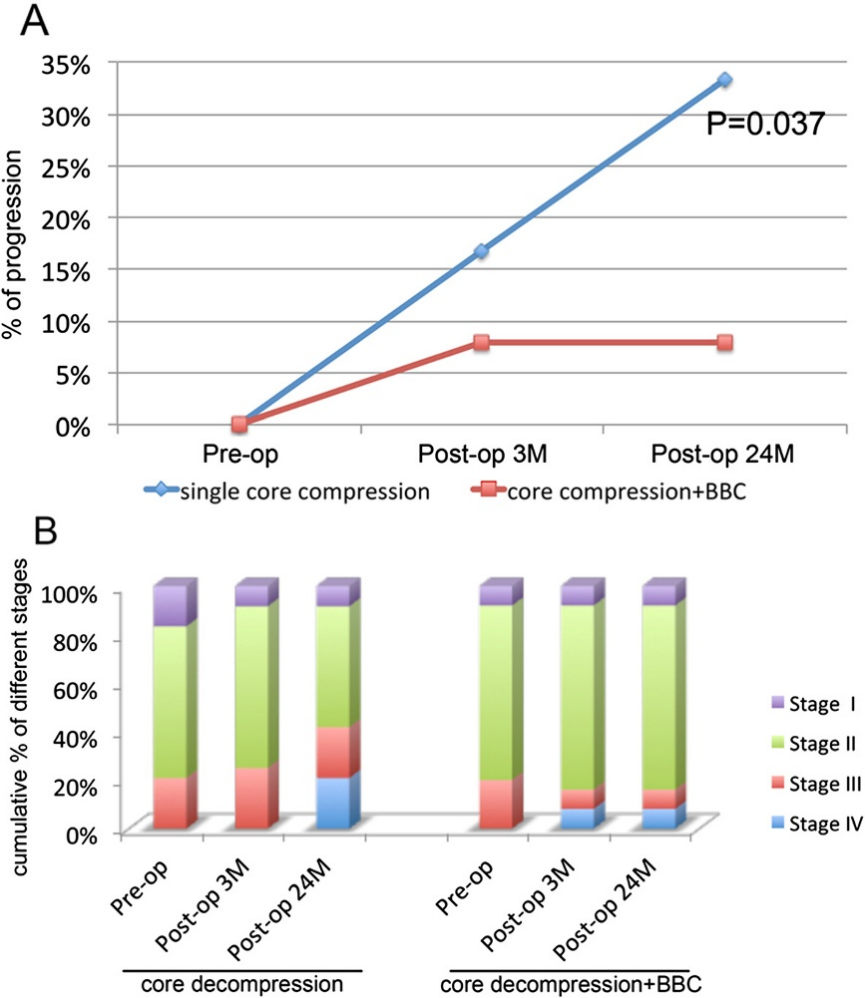
**Long-term follow-up of disease progression. (A)** Rate of progression for the control group and bone-marrow buffy coat (BBC) group of different stage avascular necrosis of femoral head (ANFH) at 3 months’ and 24 months’ follow-up. **(B)** Cumulative percentage of different Ficat stages of hip in the control and BBC group at pre-operation, 3 months post-operation, and 24 months post-operation.

At the 3 months’ follow-up of core decompression group, two hips were classified from Ficat stage I to stage II, and six hips in Ficat stage II developed into stage III/IV (Figure 4B). At the last follow-up, four patients had undergone total hip replacement surgery in the control group. The progress rates in the control group were 16.6% (4/24) at 3 months’ follow-up and 33.3% (8/24) at 2 years (Figure 4A). In the group that received core decompression + BBC implantation, only two hips had noted aggravation after treatment (Figure 4B). Both of them had progressed from Ficat stage III to stage IV and were subjected to total hip replacement. The progress rate in the treatment group was 8% (2/25) at 3 months’ and 2 years’ follow-up (Figure 4A). Most importantly, the progression rates for early-stage (I/II) hips were 0% in the treatment group and 33.3% in the control group.

## Discussion

This randomized, prospective, double-blinded cohort controlled study was designed to test the clinical outcome of BBC grafting combined with miminal invasive core decompression on patients with ANFH. Both pain relief and hip joint functional improvement were documented following the combined BBC implantation and core decompression after 3 months and 2 years. This combined strategy also reduced the disease progression of ANFH compared with core decompression without BBC implantation, especially when treated at the early stage (Ficat stage I/II). This strategy is proven to be safe, minimally invasive, and effective for the treatment of ANFH, especially at an early stage.

In this study, we conducted a 24 months’ follow-up on the patients, as collapse of femoral head is usually found in this period [15, 16]. Several classification systems have been introduced to determine the stage of the disease, including those of Ficat and Arlet, University of Pennsylvania, Association Research Circulation Osseous, and the Japanese Orthopaedic Association [16]. To be comparable to similar studies, we adopted the most widely used classification system: Ficat and Arlet [15]. In this study, patients with Ficat stage I to III ANFH were enrolled for treatment. No femoral head with early osteonecrosis (Ficat I/II) was found to have further deterioration at 24 months’ follow-up after combined core decompression and BBC implantation, whereas two out of five femoral heads with Ficat III ANFH progressed to collapse. There is a significant difference in relief of pain and improvement of hip joint function following the combined BBC implantation and core decompression after 3 months and 2 years. Although the number of Ficat III patients was limited in this study, it is suggested that the stage of ANFH might determine the success rate of bone marrow implantation. Early diagnosis and treatment might improve the clinical outcome using this combined approach.

ANFH is one of the devastating causes of disability in young patients. Early treatment of the disease should aim to prevent femoral head collapse and rescue hip joint function. Several treatment strategies have been tested to save the femoral head prior to collapse, including pharmacotherapies, physiotherapies, core decompression, vascularized or non-vascularized bone-grafting, and osteotomy [2, 4, 17–19]. As mentioned, single core decompression is the most common procedure applied because it is minimally invasive. In addition, core decompression has been proven to be partially effective in preserving femoral head collapse by reducing the intraosseous pressure and regenerating circulation that are beneficial to the disease. However, the efficacy of this technique has been variable. Particularly, single core decompression using 8 to 10 mm trephine might further deprive the subchondral support and accelerate femoral head subsidence. Small-diameter core decompression was developed to reduce cartilage damage and subchondral fracture [1]. However, the clinical failure rate of this technique still reached 30% in Ficat stage I/II patients [3, 5]. Thus, core decompression is minimally invasive but still has a high failure rate without bone grafting.

To reduce the failure rate, we modified the technique by using the autologous bone graft obtained through the trephine. The implantation of these bone tissues could enhance subchondral support, thus restraining further collapse. This has been shown by our computed tomography scanning of the femoral head that demonstrated the necrotic site was filled with a substance of similar density to bone after surgery, which was sustained for at least 6 months. This modified technique is different from other grafting techniques, which normally use vascularized or non-vascularized bone-grafting to provide a better subchondral support. However, the harvest of bone grafting from either fibula bone or iliac bone is very invasive and has complications such as donor site morbidity or nerve injury. The use of autologous bone from femoral head can avoid these side effects and provide some mechanical support in the femoral head.

In addition to mechanical support, the autologous bone graft is also an ideal biomaterial for loading cells to enhance bone regeneration in the present study. The bone graft provides an ultimate micro-environment for cell anchoring and attachment, retaining the maximal number of implanted cells. Moreover, the healthy bone graft would support the proliferation and differentiation of osteoprogenitor cells, facilitating bone formation. Indeed, accumulating evidence suggested that impaired bone formation of ANFH was caused by the reduction of focal mesenchymal stem cell pool and the number of osteoprogenitor cells [7–9]. Enriched bone marrow contains pluripotent stem/progenitors, which may contribute to both bone formation and blood vessel reconstruction in bone defect [15, 20, 21]. A limited number of randomized controlled studies of autologous bone marrow grafting for early ANFH have been carried out [15, 22–25]. The result of a prospective study of autologous bone marrow grafting on 189 femoral heads revealed that the treatment was efficient for preventing the progression of ANFH [23]. A comparison study by Gangji and colleagues [15] showed that the patients who received bone marrow mononuclear cell implantation had a significant reduction in pain and joint symptoms compared with the control group. The progression rate decreased from 62.5% in the control to 10% in the bone marrow graft group at 2 years’ follow-up [15]. Five years’ follow-up of the study further confirmed that the technique was effective for the treatment of stage I and stage II osteonecrosis [26]. Furthermore, besides containing mesenchymal stem cells, the human BBC contains various types of progenitor cells and growth factors, including endothelia progenitor cells, vascular endothelial growth factor, transforming growth factor-beta, and basic fibroblast growth factor [27]. The endothelia progenitor cells play an essential role in angiogenesis and could correct the ischemia situation in osteonecrotic lesion. The growth factors might further enhance bone regeneration. In agreement with the current literature, our study has shown that core decompression combined with the autologous bone marrow grafting in the necrotic site of femoral head might be effective for the treatment of ANFH, particularly at the precollapse stages (Ficat stage I/II).

Thus, our strategy integrated the advantages of core decompression, autologous bone graft, and BBC implantation to conduct this prospective double-blinded controlled clinical trial. This strategy is relatively feasible and favored by the clinicians. The BBC containing enriched population of mononuclear cells and cytokines could be directly loaded onto the autologous bone graft from femoral head, which can be implanted back immediately without in vitro cell expansion [28]. The clinicians could harvest and transplant the cells during the same surgical procedure and bypass the time-consuming process of cell proliferation and differentiation. The efficacy of the technique is supported by our primary outcome of pain relief and secondary outcome of hip joint function improvement. In addition, this is supported by the new bone formation in the femoral head, indicated by increased focal bone density.

There are some limitations of the study. The etiology and Ficat stages of ANFH were diverse in the participants, and the subclass patient number was limited. The number in each subclass was not able to reach efficient calculation power, and thus we did not perform statistical analysis on each subclass, and so further studies with a larger sample size and a longer follow-up period will be required. Although it has been suggested that the treatment outcome of osteonecrosis may be influenced by the cause and the stage, the numbers of each subclass between two groups was comparable. Thus, it is suggested that the implantation of BBC does have beneficial effect for the treatment of ANFH at an early stage, regardless the diverse etiological factors. However, further studies with better control on etiological factors and stage of disease will be required. Moreover, to monitor whether the implanted cells remained at the site of implantation, cell labeling will have be adapted; however, this proposal was not approved by the ethics committee. Lastly, in this study, patients were recruited according to clear enrolment criteria; however, we did not exclude the patients with bilateral ANFH. Therefore, there might be a potential contralateral effect associated with the cell treatment.

In conclusion, this study demonstrated that the combined approach of core decompression and BBC grafting could be used to prevent femoral head collapse in early-stage ANFH at 2 years’ follow-up. The result highlighted the positive effect of enriched bone marrow cells in the regeneration of focal bone necrosis. Further investigations need to be carried out to seek treatment strategies for pre-osteoarthritis ANFH after subchondral bone collapse.

## Conclusion

The present study showed that the implantation of the autologous BBC grafting combined with core decompression is effective to prevent further progression for the early stages of ANFH.

## Abbreviations

ANFH: avascular necrosis of femoral head
BBC: bone-marrow buffy coat
k-wire: Kirschner wire
VAS: visual analogue scale
WOMAC: Western Ontario and McMaster Universities Arthritis Index.
